# Echocardiographic assessment before and after Percutaneous Transvenous Mitral Commissurotomy in patients with Rheumatic Mitral Stenosis

**DOI:** 10.12669/pjms.37.1.2446

**Published:** 2021

**Authors:** Adnan Khan, Ihtesham Shafiq, Muneeb Jan, Zair Hassan

**Affiliations:** 1 Adnan Khan Department of Paediatric, Rehman Medical Institute, Peshawar, Pakistan; 2 Ihtesham Shafiq Department of Internal Medicine, Rehman Medical Institute, Peshawar, Pakistan; 3 Muneeb Jan Department of Internal Medicine, Khyber Teaching Hospital, Peshawar, Pakistan; 4 Zair Hassan Department of Cardiology, Lady Reading Hospital Peshawar, Peshawar, Pakistan

**Keywords:** Mitral Stenosis, Rheumatic fever, Mitral Commissurotomy

## Abstract

**Objectives:**

To determine the changes produced in mitral valve morphology after Percutaneous Trans-Venous Mitral Commissurotomy.

**Methods:**

Patients with mitral stenosis who underwent PTMC at the cardiology department of Lady Reading Hospital, Peshawar, Pakistan, from 2006-2016 were included in this study. All the data were manually obtained from the electronic medical record (M.F.E.). Wilkin’s echocardiographic scoring system was used to assess the severity of mitral valve thickness, leaflet mobility, valvular calcification, and Subvalvular disease. The student t-test was used for mean comparison. P-value < 0.05 was considered significant.

**Results:**

Of the total 229 patients, males were 96(41.9%), and females were 133(58.1%). The mean [SD] age of the patients was 25 ± 11years. The total Wilkin score was 7 ±1.5. 151(65.9%) were in New York Heart Association (NYHA) functional class III, and 78(34.1%) were in NYHA class IV. There was no immediate change after PTMC in systolic myocardial velocities (SV) measured at the lateral tricuspid annulus. The 2D mitral valve area increased from 0.98±0.94 cm^2^ to 1.78 ± 0.44 cm^2^ (P=0.001). Left Atrium diameter was 5.16±0.75 mm prior to PTMC, significantly decreased to 4.7± 0.7 mm (p=0.005) after PTMC. Ejection fraction (Left Ventricular Ejection Fraction) changed from 60.45± 8.25 mm Hg to 62.76±10 mm Hg (p=0.001). Mean Right Ventricular Ejection Fraction (RVEF) of patients before PTMC was 48.7 ± 4.7%, did not change significantly immediately after PTMC.

**Conclusion:**

PTMC is associated with significant changes in mitral valve morphology in terms of splitting of the fused mitral commissures, increased MVA, improved leaflet excursion, and splitting of the subvalvular structures.

## INTRODUCTION

One of the most well-known cardiac complications of rheumatic fever is mitral stenosis. Rheumatic heart disease (RHD) is still quite common in the world. The prevalence of the disease was estimated to about 33million cases worldwide with mortality of 275,000 cases annually by the global burden of disease in 2013.^1^ In other studies, conducted the prevalence calculated to be 62-78 million based on echocardiographic data.^2^,^3^ There are different forms of treatments available to the patients nowadays including medical, surgical and Per Cutaneous Transvenous Mitral Commissurotomy (PTMC) which depend on the seriousness of the symptoms, type, and severity of mitral valve stenosis and the morphology of the mitral valve.^4^

Percutaneous Transvenous Mitral Commissurotomy (PTMC) started as a new technique that enabled the mitral valve commissurotomy without the need for thoracotomy in 1984 by a Japanese cardiologist Kanji Inoue. It is being used as an alternative to thoracotomy in the treatment of mitral stenosis throughout the world.^5^ A successful PTMC causes an increase in the optimal mitral valve area (MVA) and does not cause noteworthy mitral regurgitation (MR) determined in the echocardiographic assessment.^6^,^7^ The vast majority of patient benefit straightaway from this procedure.^8^

An increase in the passion for minimally invasive procedures has aided in broadening the spectrum of PTMC where the procedure has now been tried in patients who were not the usual candidates for PTMC and with otherwise a higher risk of surgical intervention, including patients with mitral stenosis secondary to calcified mitral valves and elderly patients.^9^ According to the 2014 AHA/ACC Guidelines for the management of patients with valvular heart disease, PTMC was endorsed as a Class I indication for patients with severe MS (mitral valve area 1.5 cm) that are symptomatic and also having a favorable mitral valve morphology, in the absence of left atrial thrombus or moderate to severe Mitral regurgitation.^10^

The candidates are selected for PTMC via echocardiography as an assessment tool for the mitral valve and its various dimensions. The immediate outcomes of this study show, along with the clinical examination, the Echocardiographic score was important in selecting patients for a successful outcome.^11^ Limited data is available on this topic. Therefore, this study was performed to define the various possible changes that are produced in mitral valve morphology after PTMC.

## METHODS

A total of 229 patients with mitral stenosis who went through Percutaneous trans-venous mitral commissurotomy (PTMC) in the Department of cardiology in Lady Reading hospital, Pakistan, from 2006-2016 were included in this retrospective cross-sectional study. Patients with rheumatic heart disease, both genders and mitral stenosis were included while all those were excluded who had mitral stenosis other that rheumatic heart disease. Data was reclaimed from the electronic medical record, including demographic characteristics, symptoms, medications, comorbidities, echocardiographic data, cardiac catheterization data, and subsequent clinical outcomes after PTMC.

All the patients had a thorough clinical and echocardiographic (2 dimensional [2D]-echo, Doppler, and color flow imaging) evaluation to gauge the severity of mitral stenosis, the morphology of the valve, and mitral regurgitation (MR). Wilkin’s echocardiographic scoring system^12^ was used to assess the severity of the mitral valve thickness, mobility of the leaflet, valvular calcification, and Subvalvular disease.

2D-echocardiography estimated the Mitral valve area with polarimetry in the parasternal short-axis view utilizing the standard technique. Trans-esophageal echocardiography (TEE) was routinely performed before Balloon Mitral Valvuloplasty (BMV) in all patients included in the study. Transthoracic echocardiogram was performed multiple times, firstly during BMV, then 24 hours after the procedure, and then at follow-up visits. The contraindications were Mitral Regurgitation of Seller’s grade more than 2. A thrombus in the left atrium detected on the transthoracic echocardiography performed before PTMC and having extensive commissural calcification. All patients had been administered antibiotic cover before the procedure, and all had been heparinized after septal dilatation. Instantly before and after PTMC, the right and left heart pressures along with the mean trans-mitral pressure gradient (TMG) were measured. Left ventricular (LV) angiogram in the 30° right anterior oblique view was completed before the procedure in all patients suspected to have more than mild mitral regurgitation. The Institutional Review Boards and Ethical Committees of the hospital approved the study protocol and related materials (Ref: 05/IRB/PGMI/LRH-2019)

### Statistical Analysis

Statistical analysis was performed via SPSS software version 22. The continuous variables were measured as mean ± standard deviation (SD). The comparison of means between the two groups was made using the independent student t-test was used. P-value < 0.05 was considered significant.

## RESULTS

Out of 229 patients, males were 96(41.9%), and females were 133(58.1%). The mean [SD] age of the patients was 25 ± 11years. A female predominance was observed in this study with a female to male ratio of 1.38:1. Total Wilkin score [SD] was 7 ±1.5. About 151(65.9%) were in NYHA functional class III, and 78(34.1%) were in NYHA class IV. The patient’s baseline characteristic is shown in Table-I.

**Table-I:** Showing characteristics of patients Mean or N (%).

Age [years]	25.20±10.54
Female	133(58.1%)
BMI [KG/M^2^]	22.6±2.42
Past PTMC	15(6.6%)
New York heart association	
Class I	0
Class II	0
Class III	151(65.9%)
Class IV	78(34.1%)
Palpitation	74(34.31%)
Dyspnea	182(79.47%)
Cerebrovascular accident	8(3.5%)
Wilkin score [mean (SD)]	
Flexibility	1.8±0.5
Thickening	2.2±0.5
Subvalvular	2±0.7
Calcification	1±0.6
Total Wilkin score[mean (SD)]	7 ±1.5

In the follow-up, on two-dimensional echocardiography, the mitral value area considerably increased from 0.98±0.94 cm^2^ to 1.78 ± 0.44 cm^2^ (P<0.001). A dangerous descent in the systolic pulmonary artery pressure was observed after PTMC from 61.47 ± 8.97 mm Hg to 46.94 ± 11.22 mm Hg (p<0.001). Left Atrium diameter was 5.16 ± 0.75 mm prior to PTMC, and significantly decreased to 4.7± 0.7 mm (p=0.005) after PTMC. Ejection fraction (LVEF) changed from 60.45± 8.25 mm Hg to 62.76±10 mm Hg (p=0.001). All the changes that occurred after PTMC are shown in Table-II. The Mean RV Ejection Fraction (RVEF) of patients before PTMC was 48.7 ± 4.7 percentage and did not change significantly immediately after PTMC.

**Table-II:** Echo finding before and after PTMC.

Variable	Pre-PTMC Mean ±SD	Post-PTMC Mean ±SD	P-value

Mitral value area	0.98 ±0.94	1.78 ± 0.44	0.0001
Mitral Regurgitation	0.69 ± 0.54	1.41 ± 0.51	0.0001
Pulmonary artery pressure (PAP)	61.47 ± 8.97	46.94 ± 11.22	0.0001
Mitral value Doppler	0.91 ± 0.123	1.87 ±0.54	0.001
Left ventricle diastolic diameter (mm)	44.4±3.1	45.1 ± 2.4	0.0001
Left ventricle systolic diameter (mm)	26.1±4.6	26.7±3.4	0.0001
Left atrial diameter (mm)	5.16±0.750	4.7± 0.7	0.005
LV EF%	60.45± 8.25	62.76±10	0.001

There was no significant change in the myocardial acceleration during isovolumic contraction (IVA) and myocardial velocity during isovolumic contraction (p=0.07 and 0.08, respectively). There was no immediate change after PTMC in systolic myocardial velocities (SV) measured at the lateral tricuspid annulus. One patient had a successful replacement of the mitral valve due to mitral regurgitation after PTMC. two of the patients developed ischemic cerebrovascular accidents secondary to systemic embolization.

## DISCUSSION

In the developing world, the most common cause of mitral valve stenosis is rheumatic heart disease.^13^ It is more common in young females having average to low socioeconomic background.^14^ Over the past year, PTMC has become an alternative to surgery in the management of mitral stenosis. PTMC is a highly successful procedure with low complications with short- and long-term improvement in the patients’ symptoms.^15^,^16^ Our study exhibits immediate results of PTMC in patients with severe MS, including the Mitral valve area, mitral valve pressure gradient, pulmonary artery pressure.

In this study the procedural success was achieved in all the patients’ almost similar findings by Liaqat Ali et al. 17 The demographic spread of our patients, similar to the data available from other countries.^1^,^3^ We matched the success rate of Arora and colleagues who studied 4,838, i.e., 99.8%.^5^

This study shows that PTMC creates significant morphological and hemodynamic changes in the mitral valve. Our study established the positive instant results of PTMC in terms of a significant increase in Mitral Valve Area 0.98 ± 0.94 to 1.78 ± 0.44 cm^2^ 44 cm^2^, almost similar finding by Nawaz T & Noor A et al. (Pre-PTMC 0.96 ± 0.90 cm to 1.81± 0.48 cm,^18^,^19^ and a significant reduction in pulmonary artery pressure (PAP) 61.47 ± 8.97 to 46.94 ± 11.22mmHg. A study conducted by Ali M et al.^20^ had similar tendencies in terms of a significant increase in the Mitral Valve Area from 0.83 to 1.53 cm^2^ and a significant decrease in the mitral valve pressure gradient (MVPG) from 27.20 to 12.88. A study from Punjab Institute of cardiology by *Ahmad* A et al.^21^ showed almost similar findings with an increase in MVA from 1.0±0.3 to 2.0±0.6 cm^2^ in patients with Echo-Sc<8. In agreement with the available data.^22^,^23^ we established that PTMC produced a noteworthy increase in the Mitral Valve Area with a significant splitting of both mitral commissures. The hemodynamic results of PTMC in our study are comparable to several other studies, in which there was a reduction in the trans-mitral pressure gradient average 18 to 6 mm Hg, and also had an average increase in the mitral valve area, from 1 to 2 cm.^2^,^24^

A statistically significant increase in the mitral valve area and decrease in mean left atrium pressure after the procedure was achieved which was again comparable to other studies.^25^,^26^ Following the 2014 AHA/ACC guidelines, PTMC has now been established as the treatment of choice in patients with severe mitral stenosis in both symptomatic and asymptomatic patients.

Although PTMC is a minimally invasive procedure, there is a number factor, which affects a successful outcome, including valve morphology, the Wilkin score. PTMC is a reasonably safe procedure with a very low rate of complication, albeit, it comprises a daunting list including stroke, cardiac tamponade, procedure-related death, and regurgitation of the mitral valve (MR> 2+).^1^,^3^ Mitral Regurgitation due to PTMC, one patient had a successful replacement of the mitral valve. Secondary to systemic embolization, two of the patients developed ischemic cerebrovascular accidents. The complications are on par with most other international studies.^27^,^28^ The total number of these complications was calculated to be cerebral emboli and cardiac perforation in 1% of patients, and 2% of the patients had a severe enough MR that required the patient to be operated upon whereas almost 15% develop minor, but still undesirable, grades of MR.^24^

### Limitations of the study

Being a retrospective study, it is mostly based on the hospital database; a follow-up study could not be included in the study. Hence, further studies regarding prospective study and follow up are recommended.

## CONCLUSION

PTMC is associated with significant changes in mitral valve morphology in terms of splitting of the fused mitral commissures, increased MVA, improved leaflet excursion, and splitting of the subvalvular structures. The improvement in leaflet excursion after PTMC is determined by several morphologic and hemodynamic changes produced in the valve. The increase in MVA improves leaflet mobility within the limit.

### Authors’ Contribution:

The topic was conceived by **Adnan Khan** and he also did the data analysis.

**MJ, ZH, IS:** did the manuscript writing and the data entry.

**AK:** did the final approval of the article and accountable for the accuracy or integrity of the work.
